# Analysis and Tracking of Intra-Needle Ultrasound Pleural Signals for Improved Anesthetic Procedures in the Thoracic Region

**DOI:** 10.3390/bios15040201

**Published:** 2025-03-21

**Authors:** Fu-Wei Su, Chia-Wei Yang, Ching-Fang Yang, Yi-En Tsai, Wei-Nung Teng, Huihua Kenny Chiang

**Affiliations:** 1 Department of Anesthesiology, Taipei Veterans General Hospital, Taipei City 11217, Taiwan; fwsu@vghtpe.gov.tw (F.-W.S.); wnteng@vghtpe.gov.tw (W.-N.T.); 2 Department of Biomedical Engineering, National Yang-Ming Chiao-Tung University, Taipei City 112304, Taiwan; kevin_yang@cino.com.tw (C.-W.Y.); f910820@gm.ym.edu.tw (C.-F.Y.); ianye.tsai@garmin.com (Y.-E.T.); 3 School of Medicine, National Yang-Ming Chiao-Tung University, Taipei City 112304, Taiwan

**Keywords:** ultrasound, pleura, signal analysis

## Abstract

Background: Ultrasonography is commonly employed during thoracic regional anesthesia; however, its accuracy can be affected by factors such as obesity and poor penetration through the rib window. Needle-sized ultrasound transducers, known as intra-needle ultrasound (INUS) transducers, have been developed to detect the pleura and fascia using a one-dimensional radio frequency mode ultrasound signal. In this study, we aimed to use time-frequency analysis to characterize the pleural signal and develop an automated tool to identify the pleura during medical procedures. Methods: We developed an INUS system and investigated the pleural signal it measured by establishing a phantom study, and an in vivo animal study. Signals from the pleura, endothoracic fascia, and intercostal muscles were analyzed. Additionally, we conducted time- and frequency-domain analyses of the pleural and alveolar signals. Results: We identified the unique characteristics of the pleura, including a flickering phenomenon, speckle-like patterns, and highly variable multi-band spectra in the ultrasound signal during the breathing cycle. These characteristics are likely due to the multiple reflections from the sliding visceral pleura and alveoli. This automated identification of the pleura can enhance the safety for thoracic regional anesthesia, particularly in difficult cases. Conclusions: The unique flickering pleural signal based on INUS can be processed by time-frequency domain analysis and further tracked by an auto-identification algorithm. This technique has potential applications in thoracic regional anesthesia and other interventions. However, further studies are required to validate this hypothesis. Key Points Summary: Question: How can the ultrasound pleural signal be distinguished from other tissues during breathing? Findings: The frequency domain analysis of the pleural ultrasound signal showed fast variant and multi-band characteristics. We suggest this is due to ultrasound distortion caused by the interface of multiple moving alveoli. The multiple ultrasonic reflections from the sliding pleura and alveoli returned in variable and multi-banded frequency. Meaning: The distinguished pleural signal can be used for the auto-identification of the pleura for further clinical respiration monitoring and safety during regional anesthesia. Glossary of Terms: intra-needle ultrasound (INUS); radio frequency (RF); short-time Fourier transform (STFT); intercostal nerve block (ICNB); paravertebral block (PVB); pulse repetition frequency (PRF).

## 1. Introduction

During thoracic regional anesthesia, it is imperative to exercise utmost caution to avoid accidental puncture of the pleura, as the intended injection site is often close to this delicate membrane [1,2,3,4,5].

Ultrasonography is commonly used to identify the pleura and relevant anatomy and to guide needle insertion and injection in real time [1,5,6,7]. The pleural features appear as sliding shimmering lines on B-mode ultrasound when the visceral and parietal pleura slide against each other during the expansion and deflation of the lung during breathing cycles [8,9,10].

Similar to other miniaturized transducers used in cardiovascular catheterization for vascular imaging [11,12,13,14], tissue identification [15], and puncture safety [16], needle-sized transducers have been developed for use in anesthetic procedures [17,18,19]. Intra-needle ultrasound (INUS) transducers have been successfully used to identify the pleura during intercostal blocks [20]. The pleural signal and adjacent intercostal muscles can be visualized using INUS at the tip of the needle in real-time along the puncture using a one-dimensional radio frequency (RF) mode ultrasound signal, which presents as a unique flickering signal [20].

Previous studies have suggested that the ultrasonic features of the pleura originate from the acoustic interaction of the soft tissue and alveoli with different impedances [10]; however, the biological and physical principles of the RF-mode flickering signal of the pleura have not been fully investigated.

To differentiate the distinct patterns between ultrasound signals obtained from the pleural tissue and other tissues, we employed the short-time Fourier transform (STFT), which involves a sequence of Fourier transforms applied to a windowed signal. The STFT provides time-localized frequency information, making it suitable for situations in which the frequency components of a signal change over time [21,22,23].

The penetration and resolution of conventional ultrasound can be affected when the target is deep beneath the skin or the patient is obese [24,25]. Echogenic needles feature laser-etched marks on the needle shaft to enhance visibility. However, studies indicate that not all echogenic needle designs uniformly improve needle visualization, particularly when the needle follows a steep puncture trajectory [26]. Therefore, INUS could serve as a valuable tool to address this unmet medical need by providing image guidance directly at the needle tip, unaffected by the distance from the skin to the deep target.

We attempted to use time-frequency analysis to characterize the pleural signal and develop an automated tool that can identify the pleura during medical procedures. We used ex vivo and in vivo animal studies to investigate the pleural ultrasound signal in this study. Validation of the signal identification was also performed during clinical regional anesthesia.

## 2. Methods

### 2.1. Common Procedures in the Thoracic Region

**Figure 1:**
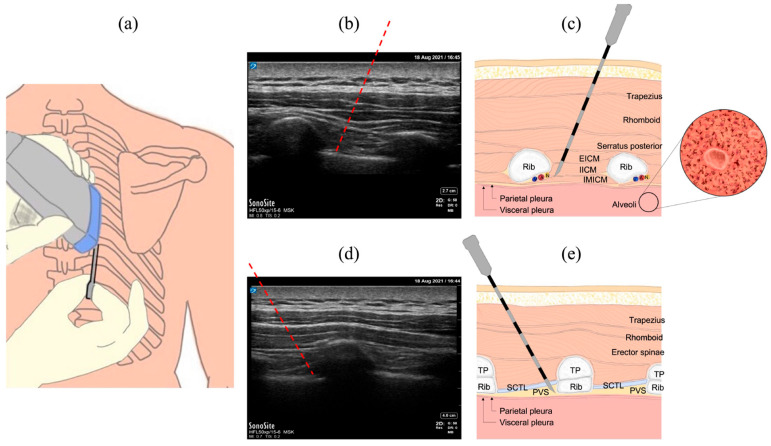
(**a**) Common procedures in thoracic anesthesia with risk of causing pleural injury and pneumothorax, (**b**) the ultrasound B-mode image, (**c**) the anatomy structure in the ICNB procedure, (**d**) the ultrasound B-mode image, and (**e**) the anatomy structure in the PVB procedure. Intercostal nerve block (ICNB); paravertebral block (PVB). The red dashed lines in (**b**,**c**) represent the proposed needle trajectories. Ultrasound images from piglets are shown in Figure 1b,d.

Figure 1a shows an intercostal nerve block (ICNB) procedure with a needle inserted into the intercostal space. The tip was placed near the intercostal nerve, which lies between the innermost and internal intercostal muscles, as shown in Figure 1b,c. In the paravertebral block (PVB) procedure, the anesthesiologist inserts a puncture needle into the paravertebral space formed among the transverse process bone, superior costotransverse ligament, and the pleura, as shown in Figure 1d,e. Local anesthetics were administered to achieve regional anesthesia.

### 2.2. The INUS System

**Figure 2:**
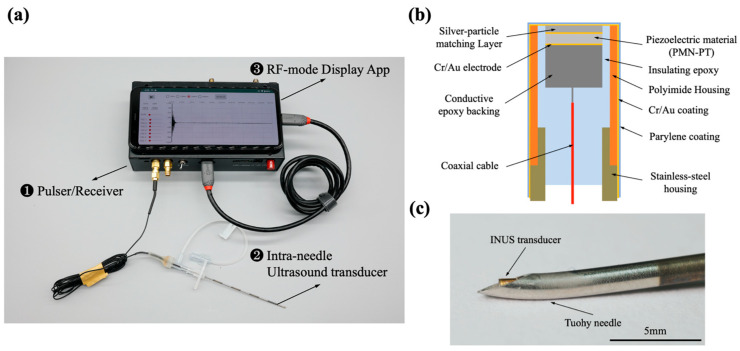
(**a**) Intra-needle ultrasound system, (**b**) the design cross-section of the needle ultrasound, (**c**) the needle transducer fit inside the Tuohy needle.

An INUS system was custom-developed to guide the induction of thoracic regional anesthesia (Figure 2a): ❶ indicates the portable ultrasound pulser/receiver, ❷ indicates the miniature INUS transducer, which was inserted into an 18-gauge puncture needle (inner diameter, 1.14 mm; outer diameter, 1.27 mm), and ❸ shows the RF-mode Display App on the Android smartphone system (Pixel 4 XL, Google Inc., Mountain View, CA, USA). The app receives and records the ultrasound signals and displays the RF mode signals on the screen of the Pixel 4 cell phone in real-time, with a pulse repetition frequency (PRF) of 30.

The needle transducer comprised a single-element lead magnesium niobate-lead titanate crystal (CTS Corp., Lisle, IL, USA). The crystal was polished to a thickness of 95 µm and cut into an area of 0.5 × 0.5 mm^2^. The design cross-section of the needle transducer is illustrated in Figure 2b. The top segment of the transducer provides a 10–15° bending flexibility, enabling the tip of the transducer to fit well in the curve-shaped anesthesia Tuohy needle (Figure 2c).

The specifications of the portable INUS system are presented in Table 1.

Calibration testing was conducted using a steel block as a reflector to evaluate performance and technical specifications. The energy intensity of the acoustic field distribution was simulated using COMSOL Multiphysics (version 5.6, COMSOL Inc., Burlington, MA, USA) in Supplemental Figure S1, which yielded a natural focus at 1.5 mm. The time-domain pulse-echo waveform and frequency-domain spectrum of the INUS transducer measured in the natural focus are shown in Supplemental Figure S2. From the Figure, the center frequency of the INUS transducer was 21.5 MHz. The fractional bandwidth at −6 dB was approximately 33%. The maximum output voltage of the pulse-echo signal was 2.04 V using the INUS system with an excitation voltage of 125 V. In our previous proof-of-concept study [20], we found the INUS system could distinguish two interfaces separated by 0.15 mm (axial resolution) and penetrate the tissue to a depth of 5 mm, and also had the ability to identify the endothoracic fascia, parietal pleura, and visceral pleura.

### 2.3. Ex Vivo Animal Study

An ex vivo phantom study was designed to simulate breathing and lung sliding to investigate if the “flickering” RF-mode pleural signal could be reproduced. A motorized platform with a resected porcine lung was designed to move back and forth to simulate the pleural sliding movement during the breathing cycle (Supplemental Figure S3a). The needle transducer was inserted into a gel pad (Parker Laboratories Inc., Fairfield, NJ, USA) and placed on top of the visceral pleural (lung) surface (Supplemental Figure S3b). Both the needle transducer and the gel pad were fixed during the lung-sliding movement. The distance between the needle tip and the visceral pleura was approximately 1.5 mm. The raw RF mode data were recorded, the PRF was set at 30 Hz, and the lung slid along the gel pad surface to simulate breathing, with a moving speed of approximately 3 cm/s, driven by the motor platform. A study on healthy subjects reported that the lung expansion distance during inspiration is approximately 4.82 ± 1.84 cm. Given that the inspiration time ranges from 1.0 to 1.2 s, a motor speed of 3 cm/s is reasonable for the simulation [27]. The motor was stopped with 0–1 s and 4–4.5 s, and the M-mode image showed a static lung image. This ex vivo animal study would also be valuable if the results demonstrate reproducibility with in vivo animal studies.

### 2.4. In Vivo Animal Study

This study was approved by the Institutional Animal Care and Use Committee of the Taipei Veterans General Hospital, Taiwan (IACUC No. 2018-96 and No. 2021-210). In this study, punctures and measurements were performed on 10 native Chinese pigs with an average weight of 25 kg [20].

**Figure 3:**
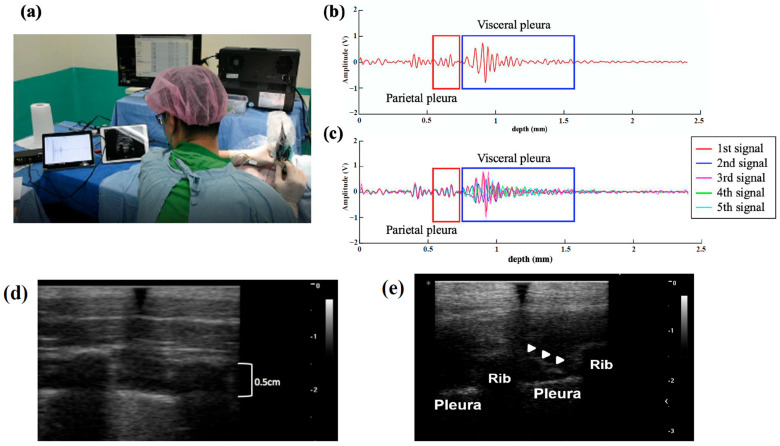
(**a**) In vivo animal study of thoracic regional anesthesia. (**b**) The RF-mode signal of the two-layered pleural membrane, the outer parietal pleura in the red box, and the inner visceral pleura in the blue box. (**c**) The overlapped plotting of five consecutive pleural signals showing the signals and flickering characteristics of the visceral pleura and alveoli during the breathing cycles. (**d**) The image obtained from surface ultrasound; the target area for injection is between ribs. (**e**) The image obtained from surface ultrasound with the needle (arrow head) in the target position of intercostal nerve block. Radiofrequency (RF).

After the induction of general anesthesia, a needle was inserted between the adjacent ribs, as shown in Figure 3a. Conventional surface ultrasound with a linear array probe was then used to confirm the insertion path and the distance from the skin to the pleura. After the skin puncture, the original stylet was replaced with an INUS transducer to guide the needle and inserted slowly toward the pleura until the pleural signal was detected 1.5 mm away from the needle tip. RF-mode ultrasound data were recorded using a 12-bit analog-to-digital converter with a sampling frequency of 125 MHz for post-analysis and M-mode image reconstruction.

The INUS transducer was excited and displayed using 30 PRF during the puncture process. Flickering pleural RF mode signals similar to pig breathing were observed on a Pixel 4 XL smartphone. Figure 3b presents the two-layered pleural signals with the parietal pleura in the red box and the visceral pleura and alveoli in the blue box. Figure 3c illustrates the overlapping RF mode signals of the pleura over five consecutive measurements during the breathing cycles. The parietal pleural layer was stationary, and the RF-mode signals consistently overlapped during the five cycles, whereas the RF-mode signals of the visceral pleura and alveoli flickered.

### 2.5. Time-Frequency Domain Analysis

An STFT analysis was used to analyze and compare the differences between the ultrasound signals of the pleural tissues and other tissues in the frequency domain. The STFT involved dividing the whole length of the acquired RF mode signals into several short depths. Then, we computed the Fourier transform for each short depth. The STFT of the ultrasound RF-mode signal *s*(*t*) can be written as

(1) $$S\left(\tau ,\omega \right)=\int_{-\infty }^{\infty }s\left(t\right)\theta \left(t-\tau \right)e^{-i\omega t}]dt$$

where *θ* (*t* − *τ*) is the window function, *τ* is the time resolution, and *ω* is the radial frequency. The transformation of the RF-mode signals was computed using MATLAB (R2020a, MathWorks Inc., Natick, MA, USA). The flow chart is presented in Supplemental Figure S4.

To quantify the flickering degree of the pleural ultrasound signal during breathing, we calculated the correlation coefficient (r) between every two consecutive pleural signals, including the RF-mode signal, M-mode image, and STFT spectrum. We also calculated the number of STFT spectral bands (−6 dB) of the RF-mode signals of the endothoracic fascia and the parietal and visceral pleura to quantify the multi-band characteristics of the ultrasonic signals from different tissues.

### 2.6. In Vivo Study of Pleura Tracking

The tracking algorithm analyzed the RF mode signal and utilized four distinct parameters to assess the region of interest (ROI) and determine its association with the pleura. These parameters are the average amplitude of the ROI, the average number of frequency bands per second, the standard deviation of the per-second frequency band counts, and the disparity between successive RF-mode data.

The signal was imported into MATLAB software and subjected to the tracking algorithm. The flowchart of pleura tracking is presented in Supplemental Figure S5.

## 3. Results

**Figure 4:**
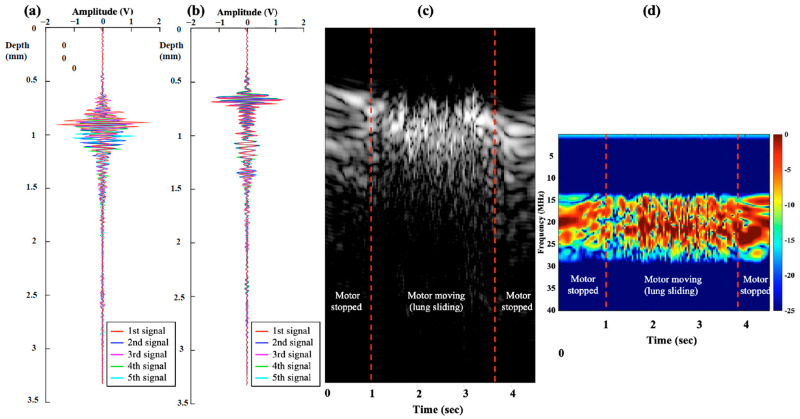
Overlapped five RF-mode signals of the ex vivo phantom when the motor was (**a**) moving (lung sliding and flickering signals) and (**b**) motor stopped (non-flickering, stable RF-signals). (**c**) M-mode image covering both the stopped and moving process. (**d**) The stable (motor stopped) and highly variable (motor moving) time-frequency spectral distribution. Radiofrequency (RF).

**Figure 5:**
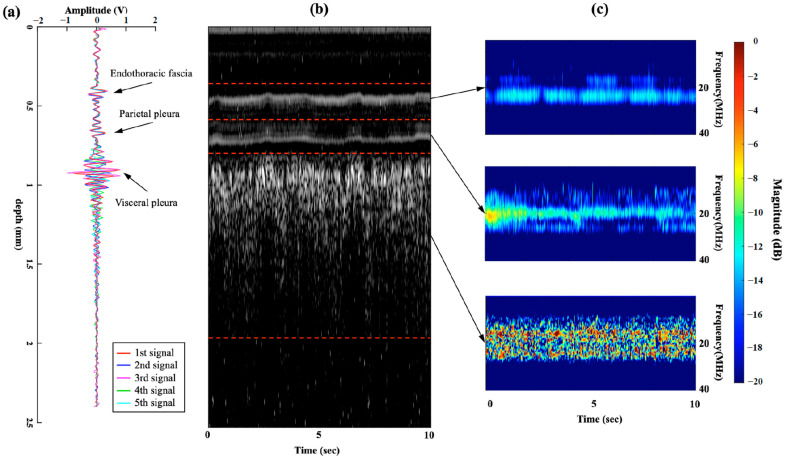
(**a**) Consecutive RF-mode signals of the endothoracic fascia and parietal pleura tissues were well overlapped, whereas those of the visceral pleura and alveoli tissues flickered in the time domain. (**b**) In five breathing cycles, the M-mode image of the endothoracic fascia and the parietal pleura was stable, whereas that of the visceral pleura and the alveoli had a speckle-like pattern. (**c**) The STFT spectra of the endothoracic fascia and parietal pleura had stable spectral bands, whereas that of the visceral pleura and alveoli were multi-banded and chaotic in the frequency domain. Radiofrequency (RF), Short-time Fourier Transform (STFT).

We observed the flickering phenomenon from the INUS-RF signals in the ex vivo and in vivo studies at the pleura during motor moving and breathing. The signals are presented in Figure 4 and Figure 5 and Supplemental Video S1.

In this figure, we aim to demonstrate the multiple and random reflections from lung tissue. When scanning a moving but relatively flat surface, the signals collected over a short period should be able to be overlaid. This indicates that the ultrasound reflections from this specific tissue are stable and do not exhibit multiple random reflections, as observed in the endothoracic fascia or parietal pleura in the latter part of our study. However, when we attempted to overlay signals from moving lung tissue collected over a short period, the signals could not be aligned. We suggest that this is caused by the random and multiple reflections from the air within the lung.

Frequency domain analysis of the returned RF signals revealed that the frequency bands of visceral pleura signals were multiple and distorted in the −6 dB frequency bands and rapidly shifted. The frequency bands of the visceral pleura were random and of mixed frequency. We speculated that this character of ultrasound waves led to the flickering phenomena observed in the RF mode and the “shimmering phenomena” in the M mode. Endothoracic fascia and parietal pleura showed stable frequency bands and fixed signals but no change in the frequency.

In the M-mode signal analysis, flickering characteristics of the visceral pleura and alveoli were also observed, with a much lower mean (m = 0.53) and higher standard deviation (σ = 0.25) of the correlation coefficient than that of the endothoracic fascia and parietal pleura tissues (Table 2). These results also validate the speckle-like characteristics in the M-mode images of the visceral pleura and alveolar tissues (Figure 5b).

Figure 5 shows the RF-mode signals, an M-mode image, and the time-frequency spectra information from the in vivo animal study. In Figure 5a, we overlapped five consecutively measured RF-mode signals from the in vivo animal study. We observed that the RF-mode signals of the endothoracic fascia and parietal pleura were stable, whereas that of the visceral pleura and alveoli flickered. In Figure 5b, we collected the M-mode image transformed from 10 s consecutively measured RF-mode signals in the in vivo animal study during five breathing cycles. The M-mode image shows two stable, bright lines representing the endothoracic fascia and parietal pleura, as well as a speckle-like region representing the visceral pleura and alveoli region. In Figure 5c, we analyzed the corresponding time-frequency spectral information covering three different depth regions. The spectral information in the 0.4–0.6 mm and 0.6–0.8 mm regions, endothoracic fascia, and parietal pleura region was stable and covered 2–3 narrower bands between 19–23 MHz. In the 0.8–2.0 mm region, the visceral pleura, and shallow alveoli region, the spectra were highly variable multi-bands and covered a significantly wider bandwidth between 12 and 28 MHz.

Table 2 shows the results of quantitative analysis with the correlation coefficient (r) analysis of fascia and pleura tissue during breathing. The M-Mode correlation coefficient for different layers is calculated by analyzing the M-Mode intensity over a continuous sequence of images within the same scanning region. With INUS set at a pulse repetition frequency of 30, there are 30 RF-mode images per second. Running STFT on these RF signals reveals multiple frequency bands in each tissue layer. We then calculate the average number of bands per second for each layer. Therefore, the higher average per second with higher standard deviation of STFT bands suggests “multiple, random, rapid changing reflections”. Table 2 compares the correlation coefficients of the STFT spectra for these tissues. The mean correlation coefficient (m = 0.39) of the visceral pleura and alveoli was lower than that of the other tissues, whereas the standard deviation (σ = 0.25) was higher.

Table 2 lists the averaged number per second of spectral bands (m = 5.5) and standard deviation (σ = 2.13) in the STFT spectra of the visceral pleura and alveoli. The average number per second of STFT bands and standard deviation of the visceral pleura and alveoli tissues were higher than that of the endothoracic fascia and parietal pleura tissues.

These results validate the chaotic (random) and multi-banded characteristics of the STFT spectra of the visceral pleura and alveoli in the frequency-domain analysis (Figure 5c).

The time-frequency spectra results of the ex vivo and in vivo studies are shown in Figure 4d and Figure 5c, respectively, revealing the multi-band and highly variable spectral characteristics of the visceral pleura and alveoli during sliding and breathing. In contrast, the spectra of the endothoracic fascia and parietal pleural signals were stable and invariant (Figure 5c), with a 0.4–0.8 mm depth region. Therefore, we can conclude that the highly variant spectral characteristics of the INUS signals were caused by lung sliding (visceral pleura and alveolar movement) during breathing.

In this study, we compared the flickering effects of visceral pleural ultrasound signals with those of the endothoracic fascia and parietal pleura (Table 2). The mean correlation coefficient of the consecutive visceral pleura and alveoli signals was significantly lower (m = 0.16) than that of the endothoracic fascia (m = 0.87) and parietal pleura (m = 0.80) signals in the RF mode. Additionally, the standard deviation of the visceral pleura and alveoli (σ = 0.33) was higher than that of the endothoracic fascia (σ = 0.13) and parietal pleura (σ = 0.16). These findings demonstrate the stable RF-mode signals of the endothoracic fascia and parietal pleura tissues and the flickering RF-mode signals of the visceral pleura and alveoli tissues (Figure 5a).

**Figure 6:**
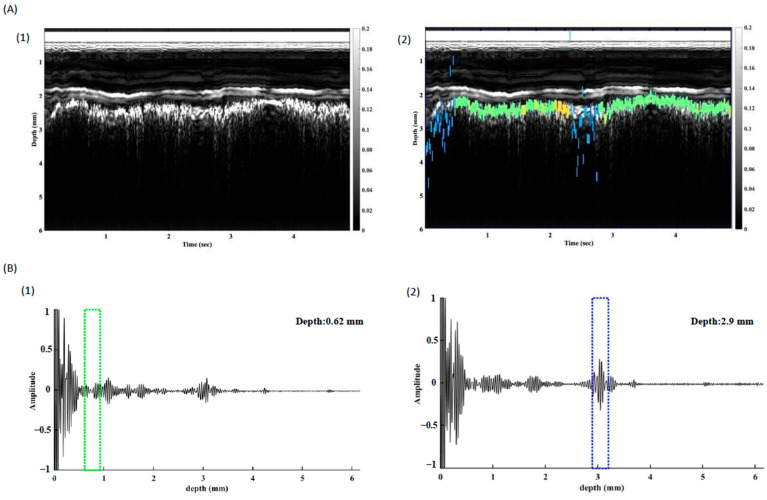
Tracking of pleura in the in vivo study. (**A**) The original (**1**) and the annotated (**2**) M-mode images from INUS. The pleura can be identified by the tracking algorithm. (**B**) A screenshot of the RF-mode video clip (Supplemental Video S2) shows the pleural auto-identification process. After applying the STFT algorithm, the green box represents a “guessed but not confirmed” status during the pleural location search. Furthermore, the blue box in the RF-mode diagram and the green line in the M-mode represent the signal that fits the pleural characteristics of our analysis and are visually highlighted for clinicians to avoid accidental puncture. Intra-needle ultrasound (INUS), Radiofrequency (RF).

The multiband count within the spectrum held considerable relevance in identifying the pleura. The recorded signal was further processed with the tracking algorithm, and the pleura was traced successfully (Figure 6). The INUS system allows users to switch between RF-mode and M-mode, improving confidence in identifying the pleura’s location during procedures.

## 4. Discussion

As our goal was to avoid pleural puncture, identifying the pleura from the needle tip, i.e., from INUS, could present a promising strategy when dealing with limited image quality from external ultrasound, such as in cases of deep punctures or in overweight patients [24]. We effectively discerned the distinct features of the pleural signal from INUS. Only the visceral pleura and alveoli showed high variability and flickering characteristics in the time-domain RF-mode signals and multibanded and chaotic characteristics in the frequency-domain spectra during respiration in the ICNB and PVB procedures. We suggest this is ultrasound distortion caused by the interface of multiple moving alveoli, and the signal returned in variable and multi-banded frequency. The RF-mode signals of the visceral pleura and alveoli showed high variability during the breathing cycle, causing a “flickering phenomenon” due to their fast changes and a frame rate of display of 30 Hz. This is due to the persistence of vision, which lasts for approximately 100 ms [28].

The “flickering phenomenon”, the speckle-like M-mode image, and the shimmering B-mode image (lung sliding sign) disappear when the lung is not breathing. We suggest that these essentially originate from the same mechanism: visceral pleura and alveoli movement during lung sliding during breathing.

Some difficulties may be encountered when using ultrasound guidance to perform thoracic regional anesthesia in patients with obesity because of the presence of subcutaneous fat, which has strong attenuation and may reduce image quality, making it difficult to accurately discern the location of the needle tip relative to the pleura. Additionally, procedures near the spine, such as PVB, can make it more challenging for ultrasound to fully delineate the pleura, as it lies deeper in this region. Inaccurate identification of the pleura or the distance between the pleura and needle tip can result in complications, such as inadvertent pleural puncture, pneumothorax, and ineffective analgesia [5,6,7,9,10,11]. Although the quality of conventional surface ultrasonic images continues to improve, accurately advancing the needle to deeply located targets remains a challenge for anesthesiologists [8].

Pleura identification was successfully performed using the INUS transducer in the ex vivo and the in vivo study, and the characteristics were successfully integrated into an auto-identification algorithm for real-time monitoring and imaging. The M-mode image can be constructed in real-time from the digitized RF-mode signals to provide a detailed display of the forward view of the INUS to guide the needle insertion process regardless of the depth of the skin or the limitations of conventional surface ultrasound.

These distinctive and specific results may provide valuable information for our group to develop a visceral pleura auto-identification algorithm. In addition, the sliding of the pleura (lungs) during breathing may differ between animals and humans. Therefore, further clinical studies are warranted.

In conclusion, the unique flickering signal of the pleura from INUS can be processed by time-frequency domain analysis and further tracked by an auto-identification algorithm. This technique can be used for thoracic regional anesthesia and other interventions. However, further studies are required to confirm this hypothesis and validation of the auto-identification of the pleura will be performed.

## Figures and Tables

**Table 1:** Specifications of the INUS system.

Type	Parameter	Value
Transducer	Type	Pulse-echo
	Number of elements	Single element
	Natural Focus	1.5 mm
Pulser	Frequency	20 MHz
	Pulse-repetition frequency	30 Hz
	Analog-to-Digital Converter	12 bits
	Sampling rate	125 MHz
	Pulse-type	Sine wave
	Excitation voltage	125 V
	Dimensions	163 mm × 90 mm × 42 mm
	Weight	670 g
	Display	Pixel 4 XL

**Table 2:** Quantitative analysis of the flickering effects of the fascia and pleura tissue signals (m ± σ).

	Endothoracic Fascia	Parietal Pleura	Visceral Pleura + Alveoli
Correlation coefficient of RF-mode signals	0.87 ± 0.13	0.80 ± 0.16	0.16 ± 0.33
Correlation coefficient of M-mode signals	0.94 ± 0.04	0.80 ± 0.15	0.53 ± 0.25
Correlation coefficient (STFT Spectra)	0.93 ± 0.07	0.85 ± 0.14	0.39 ± 0.25
Average number per second of STFT bands (m ± σ)	2.7 ± 0.84	2.7 ± 0.81	5.5 ± 2.13

Mean (m); standard deviation (σ); radiofrequency (RF); short-time Fourier transform (STFT).

## Data Availability

Data supporting the findings of this study are available from the corresponding author HK Chiang on request.

## Supplementary Materials

The following supporting information can be downloaded at: https://www.mdpi.com/article/10.3390/bios15040201/s1, Figure S1: Simulated Acoustic Field Energy Intensity; Figure S2: Time-Domain Pulse-Echo Waveform and Frequency-Domain Spectrum of the INUS Transducer; Figure S3: Platform of the Ex Vivo Animal Study; Figure S4: Flowchart of the STFT Analysis; Figure S5: Overview of the Pleural Tracking Algorithm Flowchart; Video S1: Pleural Flickering Signal Observed in INUS-RF Signals; Video S2: Video Demonstration of Needle Tip–to–Pleura Distance Estimation Using INUS-RF Signal Tracking.
