# Supplementary material for: Analysis and Tracking of Intra-Needle Ultrasound Pleural Signals for Improved Anesthetic Procedures in the Thoracic Region
Source: Biosensors (Basel). 2025 Mar 21;15(4):201. doi: 10.3390/bios15040201 (PMC12025225; doi:10.3390/bios15040201)
Supplement: Supplementary file 1 [file biosensors-15-00201-s001.zip › Supplemental Figure S2.pdf]

Supplemental Figure S2: Time-Domain Pulse-Echo Waveform and Frequency-Domain Spectrum of the INUS Transducer

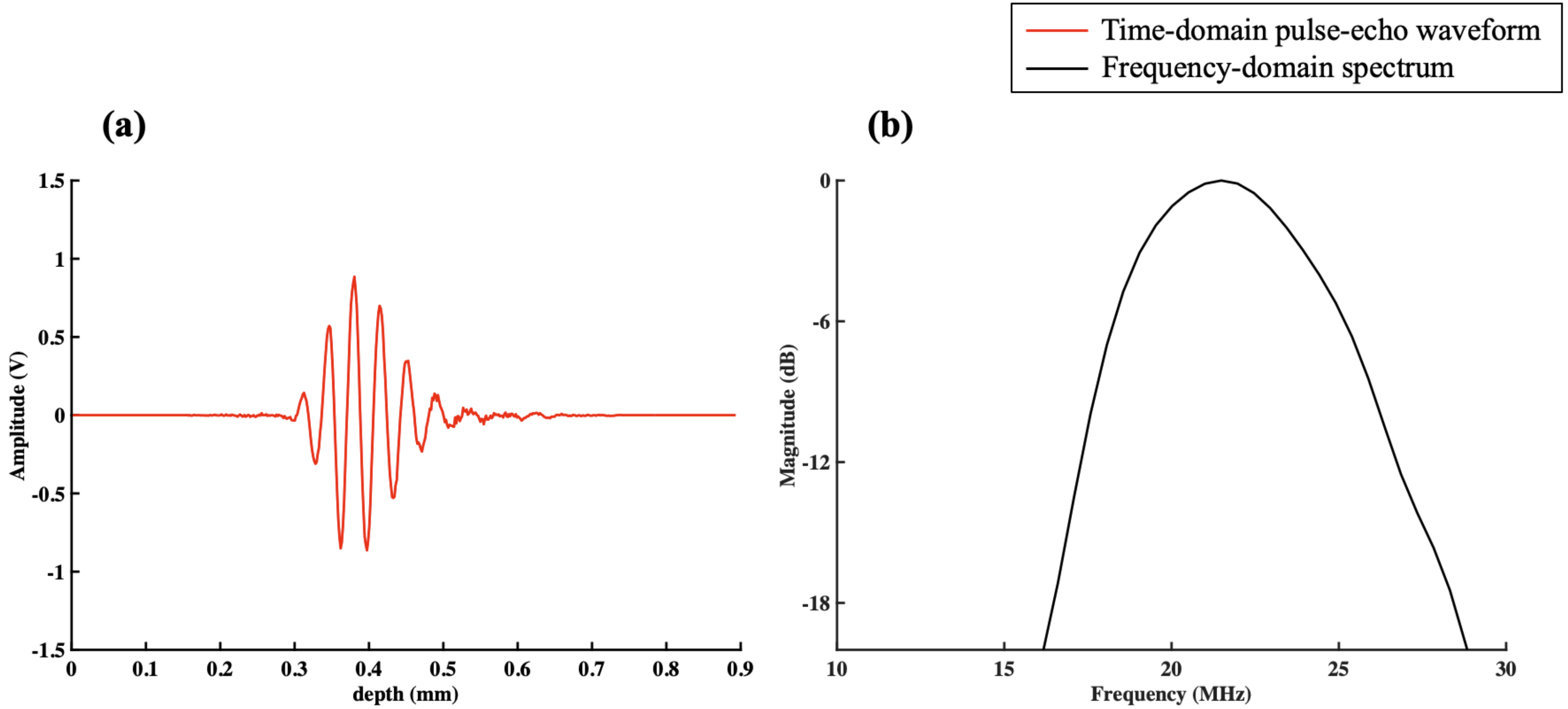

Figure S2. (**a**) Time-Domain Pulse-Echo Waveform, (**b**) Frequency-Domain Spectrum
