# Supplementary material for: Analysis and Tracking of Intra-Needle Ultrasound Pleural Signals for Improved Anesthetic Procedures in the Thoracic Region
Source: Biosensors (Basel). 2025 Mar 21;15(4):201. doi: 10.3390/bios15040201 (PMC12025225; doi:10.3390/bios15040201)
Supplement: Supplementary file 1 [file biosensors-15-00201-s001.zip › Supplemental Figure S3.pdf]

Supplemental Figure S3. Platform of the Ex Vivo Animal Study

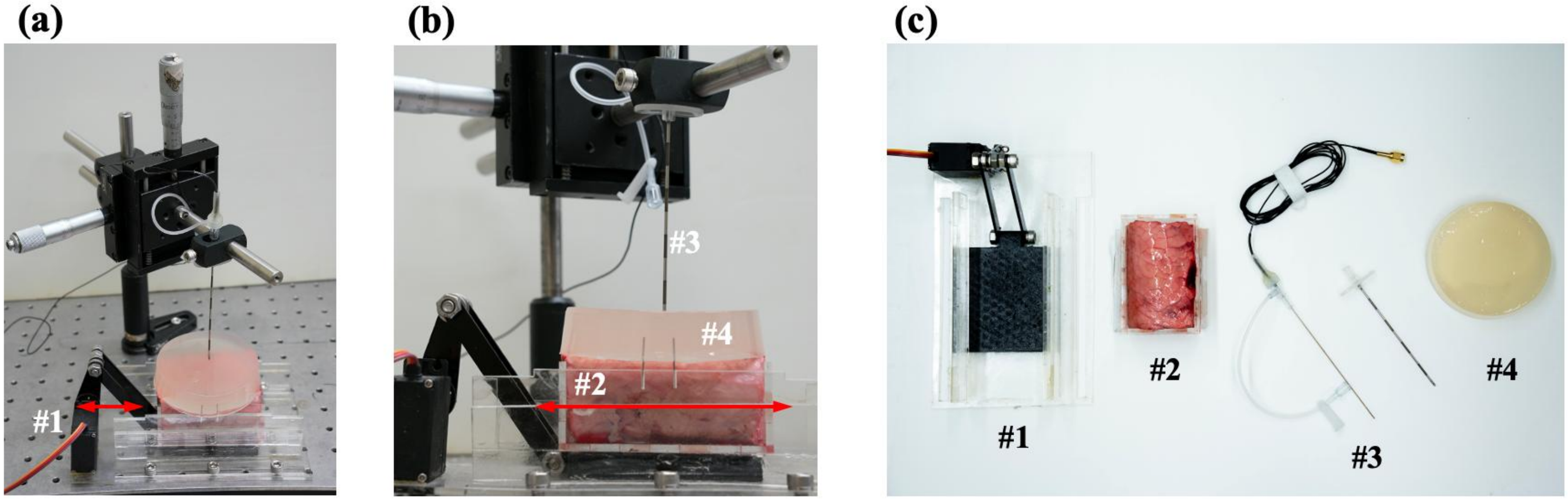

Supplemental Figure S3. (a) Oblique view of the full phantom showing #1 the motor platform; (b) front view of the phantom showing #2 the porcine lung tissue in a box, #3 the INUS transducer and the Tuohy needle, and #4 the fixed gel pad. Arrows point the direction of lung sliding. (c) the four parts of the phantom, exploded view.
