# Supplementary material for: Analysis and Tracking of Intra-Needle Ultrasound Pleural Signals for Improved Anesthetic Procedures in the Thoracic Region
Source: Biosensors (Basel). 2025 Mar 21;15(4):201. doi: 10.3390/bios15040201 (PMC12025225; doi:10.3390/bios15040201)
Supplement: Supplementary file 1 [file biosensors-15-00201-s001.zip › Supplemental Figure S4.pdf]

Supplemental Figure S4: Flowchart of the STFT Analysis

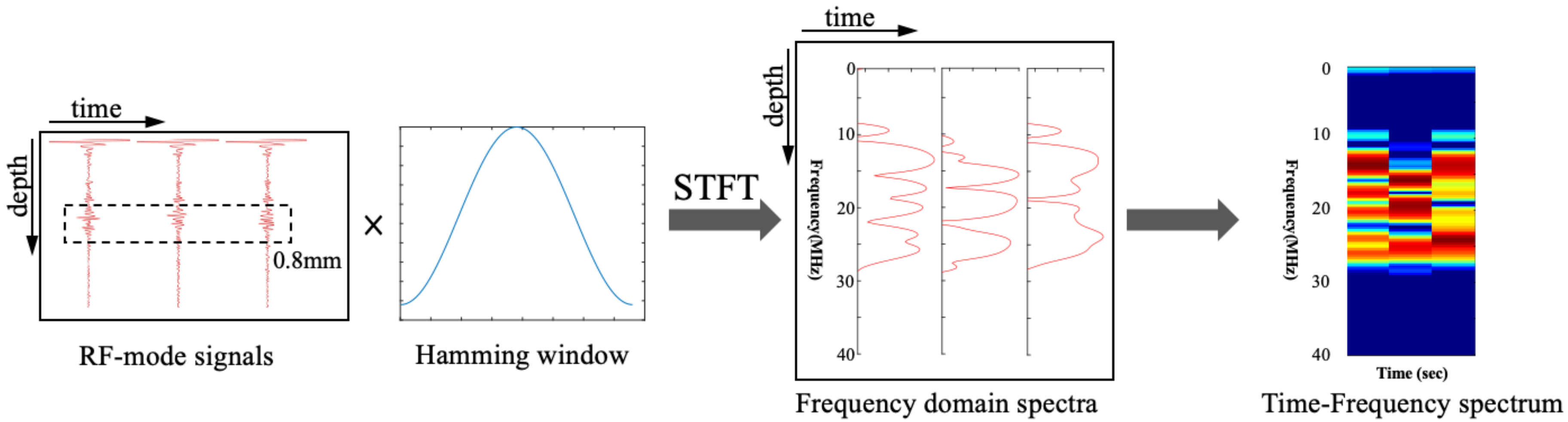

Supplemental Figure S4: Flowchart of the STFT Analysis

The procedure of the Time-Frequency spectrum by combining the STFT of the RF-mode signal at the certain depth. STFT: short-time Fourier transform.
