# Supplementary figures and images for: Analysis and Tracking of Intra-Needle Ultrasound Pleural Signals for Improved Anesthetic Procedures in the Thoracic Region
Source: Biosensors (Basel). 2025 Mar 21;15(4):201. doi: 10.3390/bios15040201 (PMC12025225; doi:10.3390/bios15040201)

Supplemental Figure S1. Simulated Acoustic Field Energy Intensity

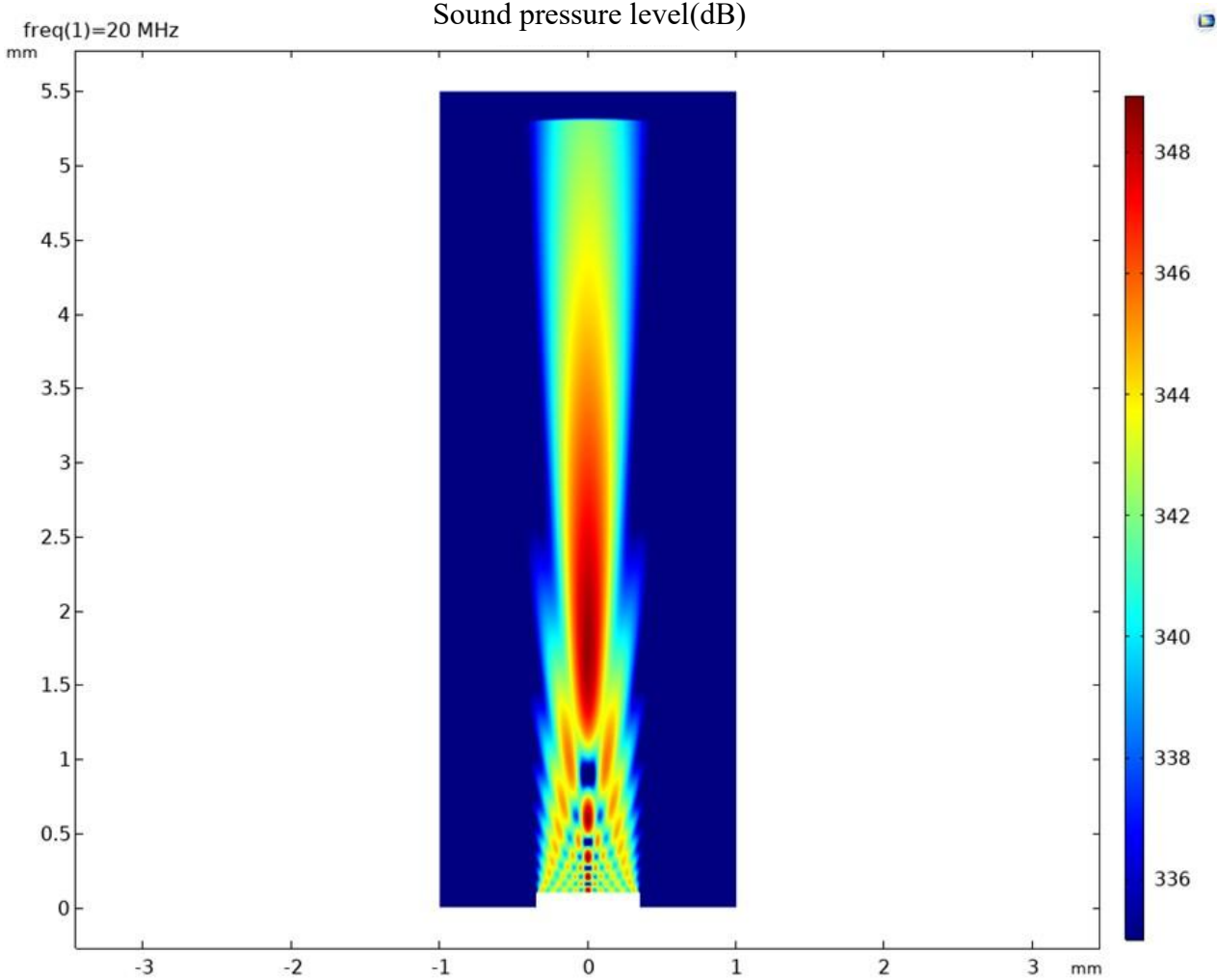

Supplement: Supplementary file 1 [file biosensors-15-00201-s001.zip › Supplemental Figure S1.pdf]

Supplemental Figure S5. Overview of the Pleural Tracking Algorithm Flowchart

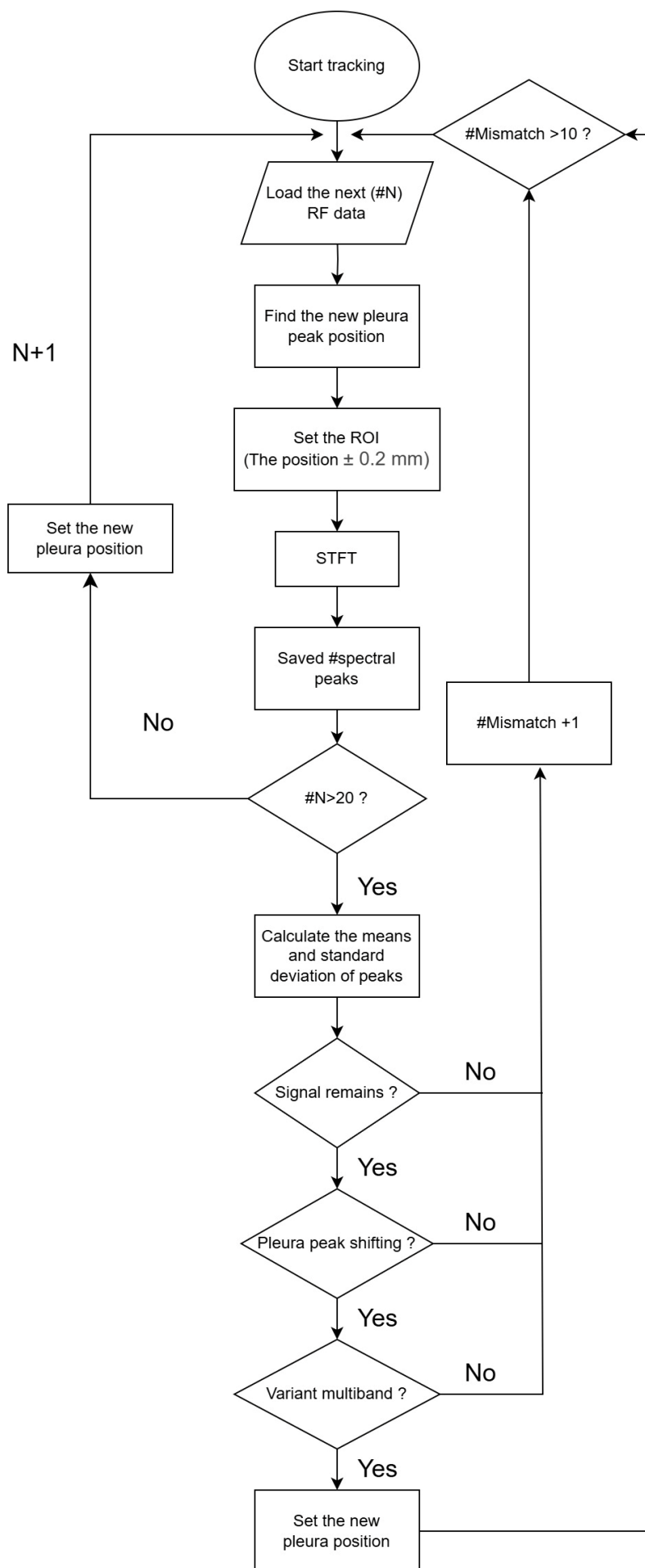

Supplement: Supplementary file 1 [file biosensors-15-00201-s001.zip › Supplemental Figure S5.pdf]
